# Auditory and Visual Health after Ten Years of Exposure to Metal-on-Metal Hip Prostheses: A Cross-Sectional Study Follow Up

**DOI:** 10.1371/journal.pone.0090838

**Published:** 2014-03-12

**Authors:** Jennifer R. Prentice, Christopher S. Blackwell, Naz Raoof, Paul Bacon, Jaydip Ray, Simon J. Hickman, J. Mark Wilkinson

**Affiliations:** 1 Department of Human Metabolism, University of Sheffield, Sheffield, South Yorkshire, United Kingdom; 2 Department of Ophthalmology, Sheffield Teaching Hospitals NHS Foundation Trust, Sheffield, South Yorkshire, United Kingdom; 3 Department of Otolaryngology, Sheffield Teaching Hospitals NHS Foundation Trust, Sheffield, South Yorkshire, United Kingdom; 4 Department of Neurology, Sheffield Teaching Hospitals NHS Foundation Trust, Sheffield, South Yorkshire, United Kingdom; Univ Rochester Medical Ctr, United States of America

## Abstract

Case reports of patients with mal-functioning metal-on-metal hip replacement (MoMHR) prostheses suggest an association of elevated circulating metal levels with visual and auditory dysfunction. However, it is unknown if this is a cumulative exposure effect and the impact of prolonged low level exposure, relevant to the majority of patients with a well-functioning prosthesis, has not been studied. Twenty four male patients with a well-functioning MoMHR and an age and time since surgery matched group of 24 male patients with conventional total hip arthroplasty (THA) underwent clinical and electrophysiological assessment of their visual and auditory health at a mean of ten years after surgery. Median circulating cobalt and chromium concentrations were higher in patients after MoMHR versus those with THA (P<0.0001), but were within the Medicines and Healthcare Products Regulatory Agency (UK) investigation threshold. Subjective auditory tests including pure tone audiometric and speech discrimination findings were similar between groups (P>0.05). Objective assessments, including amplitude and signal-to-noise ratio of transient evoked and distortion product oto-acoustic emissions (TEOAE and DPOAE, respectively), were similar for all the frequencies tested (P>0.05). Auditory brainstem responses (ABR) and cortical evoked response audiometry (ACR) were also similar between groups (P>0.05). Ophthalmological evaluations, including self-reported visual function by visual functioning questionnaire, as well as binocular low contrast visual acuity and colour vision were similar between groups (P>0.05). Retinal nerve fibre layer thickness and macular volume measured by optical coherence tomography were also similar between groups (P>0.05). In the presence of moderately elevated metal levels associated with well-functioning implants, MoMHR exposure does not associate with clinically demonstrable visual or auditory dysfunction.

## Introduction

More than 500,000 patients in the United States have received metal-on-metal hip prostheses, including metal-on-metal hip resurfacing (MoMHR), the majority of whom have well-functioning devices [1]. Cobalt and chromium are the principal metals released by MoMHR [2], [3]. Ten-year steady state median blood cobalt and chromium concentrations in patients with well-functioning devices are between 1.5 and 2.3 µg/L, but remain 10-fold higher than physiological levels [4], [5].

Case reports relating to prosthesis failure commonly include visual and auditory problems associated with grossly elevated blood metal concentrations [6], [7], [8], [9], [10], [11]. Tower et al. described two cases in which prosthesis failure associated with hearing loss [10]. Ikeda et al. quantitated hearing loss by pure tone audiometry and demonstrated hearing thresholds of 52 and 45 dB in the right and left ears respectively (average over 0.5, 1, 2 and 4 kHz, normal <25) [11]. The patient described by Rizzetti et al. had bilateral absence of brainstem acoustic responses [8]. Assessment of visual loss within the case studies showed in one case undefined visual changes and hyper-intensity of the optic nerves and tracts on magnetic resonance imaging (MRI), and ‘irregular’ cortical visual responses when measuring visual evoked potentials in another subject. Whilst these cases highlight association between overt failure of prosthetic devices and auditory or visual dysfunction, the effect on these sensory systems of prolonged exposure to moderately elevated blood metal concentrations, relevant to the great majority of patients with these devices, has not been defined.

Between 2009 and 2010 we undertook a single-centre cross-sectional study to investigate the systemic effects of chronic metal exposure following MoMHR [12]. Thirty-five patients after MoMHR were individually matched by age, sex and time since surgery to patients who had received a conventional total hip arthroplasty (THA) using a non-metal-on-metal bearing at a mean of 8 years following surgery. Patients within both groups were in good self-reported health and had well-functioning prostheses. We identified differences in grey matter density in the occipital cortex and in the cross-sectional area of the optic chiasm in subjects receiving MoMHR versus those who had received a conventional THA, indicating an association between MoMHR exposure and subtle structural changes in the visual system of the brain [13]. Following these findings we recalled all 62 male participants in the study order to conduct a detailed auditory and visual assessment to determine whether chronic low elevated metal exposure in well-functioning MoMHR associates with demonstrable functional effects on these systems.

## Patients and Methods

### Ethics, design, setting, and participants

This extension study was approved by South Yorkshire Research Ethics Committee, and all patients provided written informed consent prior to participation. This single centre study was undertaken as an extension of a cross sectional study Investigation examining the systemic effects of metal exposure in MoMHR. In this extension study all men (n = 31 MoMHR and 31 age-matched THA patients) were invited to return for further visual and auditory assessment. Patients were recruited from the community between November 24^th^, 2009 and May 20^th^, 2010. The study design was reviewed by a statistician at Sheffield Clinical Trials Research Unit, and its conduct and dissemination of results were reviewed by Sheffield Lay Advisory Panel for Bone Research. The study inclusion and exclusion criteria are documented elsewhere [12]. Blood and plasma cobalt and chromium were measured by inductively-coupled plasma-mass spectroscopy (ICP-MS), as described previously [12]. Briefly, blood samples were collected via plastic cannulae using the last draw method directly into trace element collection tubes, and metal assays were performed by a laboratory that participates in national trace element measurement quality assurance schemes (TEQAS).

### Auditory Outcomes

#### Clinical evaluation

All subjects were questioned about their auditory health including occupational noise exposure, tympanic membrane perforation, tinnitus and current respiratory infections. Otoscopic examination was performed by an otolaryngologist to determine the presence of any co-existent pathology. All Audiometric assessments were made in sound attenuated test rooms.

#### Subjective audiometric tests

Pure tone audiometry (PTA) was performed in accordance with the British Society of Audiology recommended procedures (The British Society of Audiology, 2011) using an Aurical Audiometer type 1081 and its associated OTO suite application software module v. 4.50.01 (GN Otometrics A/S). Air conduction (AC) thresholds were obtained using TDH39 headphones across the frequency range 0.25 to 8 kHz. If AC thresholds were raised above 20 dB hearing level (dBHL) then bone conduction thresholds were measured using a B-71 bone oscillator across frequencies 0.5 to 4 kHz. The integrated speech test module was used with the AB word lists and the 50 per cent speech recognition level measured for each ear [14].

#### Objective audiometric tests

Evoked oto-acoustic emissions were recorded to assess cochlear function whilst the electrophysiology tests of auditory brainstem response (ABR) and the auditory cortical response (ACR) were used to assess for retro-cochlear pathology in the auditory brainstem and auditory cortex respectively. Both Transient evoked otoacoustic emissions (TEOAEs) and distortion-product oto-acoustic emissions (DPOAEs) were recorded using a dual probe DP Echoport (ILO292-II USB interface) coupled with a laptop using the ILOv6 software (v 6.34.22.0). For the TEOAEs, non-linear type stimuli with a target level of 84 dB SPL were presented. Noise rejection threshold was set to 40 dB SPL and 360 sweeps were averaged. The resulting TEOAEs were analysed over a 2.5 to 20 millisecond time window and in half octave bands OAE power (1, 1.4, 2, 2.8 and 4 kHz). A response was judged to be present in a given half octave band if the signal was >−5 dBSPL with a signal to noise ratio >6 dB. For the DPOAEs, two primary tones at frequencies f_1_ and f_2_ were presented at levels L_1_ and L_2_ = 70 dB SPL with a fixed frequency ratio of f_2_/f_1_ at 1.22.

Oto-neurologic auditory brainstem evoked responses (ABRs) were recorded using a Biologic Navigator Pro AEP system. Subjects were asked to relax and if possible go to sleep in a reclined chair in order to minimise myogenic and movement artifacts. Alternating polarity 100 µs duration clicks at a repetition rate of 11.1 per second were presented at a stimulus level of 80dBnHL using TDH-39P headphones. Two channel evoked potential activity was recorded. Electrodes were placed on the forehead (common), vertex (jumpered to both channels) and on the left and right mastoids. EEG activity was sampled over 512 points for a 10.66 ms time epoch (0.5 ms blocking) and amplified with a gain setting of 150000. Filtering was from 100 to 3000 Hz with an artefact rejection level of ±10 µV peak to peak. A minimum of two replicated averages each with a minimum of 1800 sweeps were recorded. The absolute ipsilateral Wave V latency for each ear was measured along with inter-aural latency differences [15]. A subjective threshold to the ABR test stimuli was also measured for each ear.

Auditory cortical evoked responses (ACR) were recorded using the same Biologic Navigator Pro AEP system and the same electrode configuration as used for ABR recordings. Subjects were sat up in a chair and asked to maintain an alert state by reading a magazine or newspaper. Rarefaction tone burst stimuli calibrated at 80 dB HL and at a frequency of 1000 Hz (10 cycle linear ramp and 50 cycle plateau) were presented at a repetition rate of 0.5 per second using TDH-39P headphones. EEG activity was sampled over 512 points for a 533 ms time epoch and amplified with a gain setting of 75000. Filtering was from 1 Hz (12 dB/octave) to 15 Hz with an artefact rejection level of ±45 µV peak to peak. A minimum of two replicated averages each with a minimum of 40 sweeps were recorded. These replications were further averaged together to form a grand average and to optimise peak identification. The P1-N1-P2 sequence was identified and the amplitudes measured for both ipsilateral and contralateral recordings. A subjective threshold to the ACR test stimuli was also measured for each ear.

### Visual Outcomes

#### Clinical evaluation

The ophthalmic evaluation comprised an ocular and targeted medical history, as well as an ocular examination made by an Ophthalmologist to exclude primary ocular pathology. All patients were examined for pupillary defects and underwent visual field testing to confrontation. Ocular motility examination, Goldmann applanation tonometry and slit lamp biomicroscopy were also undertaken. The dilated fundus was then examined by slit lamp biomicroscopy using 78D and 90D lenses.

#### Visual symptoms

The National Eye Institute Visual Functioning Questionnaire (NEI-VFQ) was used to measure self-reported visual function and general health implications [16]. The 10 question neuro-ophthalmic supplement was administered to identify visual defects due to neurological disorders [17]. These were administered before the rest of the evaluation and vision testing to avoid bias.

#### Subjective visual tests

Uniocular and binocular best corrected logMAR visual acuities were measured using Early Treatment Diabetic Retinopathy Study (ETDRS) charts at 4 m. Binocular low contrast acuity was measured using 4 m ETDRS charts of 1.25% and 2.5% contrast and recorded as logMAR acuity. Colour vision was assessed binocularly using the Farnsworth-Munsell 100-hue test (Munsell Color Company, Grand Rapids, MI, USA) administered under simulated daylight (20 W Compact fluorescent light of 1152lm and 65,000K colour temperature) with no time limit imposed. Total error scores were calculated for each participant using the traditional scoring method [18]. Partial error scores for the blue-yellow colour axis and the red-green axis were also calculated for each participant. A square root transformation was used to normalize the distribution of error scores for the statistical analyses.

#### Objective visual tests

Retinal nerve fibre layer thickness and macular volume were then measured using Spectralis optical coherence tomography (OCT) (Heidelberg Engineering Ltd, Heidelberg, Germany). In both eyes, retinal nerve fibre (RNFL) thickness was measured using the Fast RNFL scanning protocol in which three circular 3.4 mm diameter scans, centred on the optic disc were taken, the mean of which was then used to express RNFL thickness. Macular volume was measured using the Fast Macular Thickness map protocol, and 6 mm by 6 mm (20° by 20°) of the macular was scanned by means of 25 vertical B scans centred on the fovea.

### Statistical analysis

Between group analyses of PTA were made in each ear independently, and speech discrimination in the best ear and as the mean value for both ears. Auditory electrophysiology data was analysed as the mean value for both ears. OCT measurements were used for one randomly selected eye from each subject in both groups unless there was pre-existing ocular disease in one eye, when the measurement from the fellow unaffected eye was used, as described by Henderson et al. [19]. Between group analyses of continuous datasets were made using Students t-test or the Mann-Whitney test, as appropriate. Categorical data were analysed using the Chi-squared test. All tests were made 2-tailed with a critical p-value of 0.05, and made using IBM SPSS statistical software (version 20, IBM, Portsmouth, UK).

## Results

### Participants and metal levels

24 participants with MOMHR and 24 with THA agreed to participate in this extension study. The groups were well matched for age and time since surgery (Table 1). Median blood, plasma, and urinary cobalt and chromium were 5 to 10 fold higher in MoMHR versus THA patients (P<0.0001). However, blood median and interquartile range metal levels were below guidance threshold concentration of 7 µg/L suggestive of prosthesis mal-function, and set by the Medicines and Healthcare Products Regulatory Agency (MHRA) in the United Kingdom (MDA/2012/008, 28 February).

**Table 1:** Patient characteristics.

Characteristic	MoMHR (n = 24)	THA (n = 24)	P-value
Age at assessment (years)	62.5±7.1	62·6±7.5	0.95
Age at time of surgery (years)	52.3±7.2	52.9±8.6	0.79
Time since surgery (years)	10.2±1·8	9.7±2·5	0.49
*Serum cobalt (µg/L)	0.47 (0.36 to 1.05)	0.06 (0.04 to 0.08)	<0.0001
*Serum chromium (µg/L)	0.61 (0.47 to 2.31)	<0.1 (<0.1 to <0.1)	<0.0001
*Whole blood cobalt (µg/L)	0.43 (0.29 to 1.26)	0.05 (0.03 to 0.07)	<0.0001
*Whole blood chromium (µg/L)	0.48 (0.33 to 1.82)	0.10 (<0.1 to 0.13)	<0.0001

Data are mean ±SD or median (interquartile range). Analyses are MoMHR versus THA by Student's t-test or Mann Whitney test.

### Auditory outcomes

#### Clinical audiological evaluation

The two groups were well matched based on audiological history findings except self-reported loud noise exposure, which was greater in the THA group with 19 cases compared to 11 in the MOMHR group (Table 2). No patients were excluded from auditory testing following clinical assessment.

**Table 2:** Audiology medical history.

Characteristic	MoMHR (n = 24)	THA (n = 24)	P-value
Self-reported hearing loss	8 (33%)	10 (42%)	0.77
Previous otalgia	1 (4%)	1 (4%)	1.00
Loud noise exposure	11 (46%)	19 (79%)	0.04
Use of hearing aids	1 (4%)	0	1.00
Current tinnitus	5 (21%)	8 (33%)	0.52
Current balance problems	0	2 (8%)	0.49
Previous tinnitus	8 (33%)	14 (58%)	0.15
Previous balance problems	3 (13%)	3 (13%)	1.00
Ear surgery	0	0	1.00
Previous perforated tympanic membrane	2 (8%)	1 (4%)	1.00
Current ear infection	0	0	1.00

Data are number of patients (percentage). Analyses are MoMHR versus THA by Fisher's exact test.

#### Subjective audiometric tests

**Figure 1:**
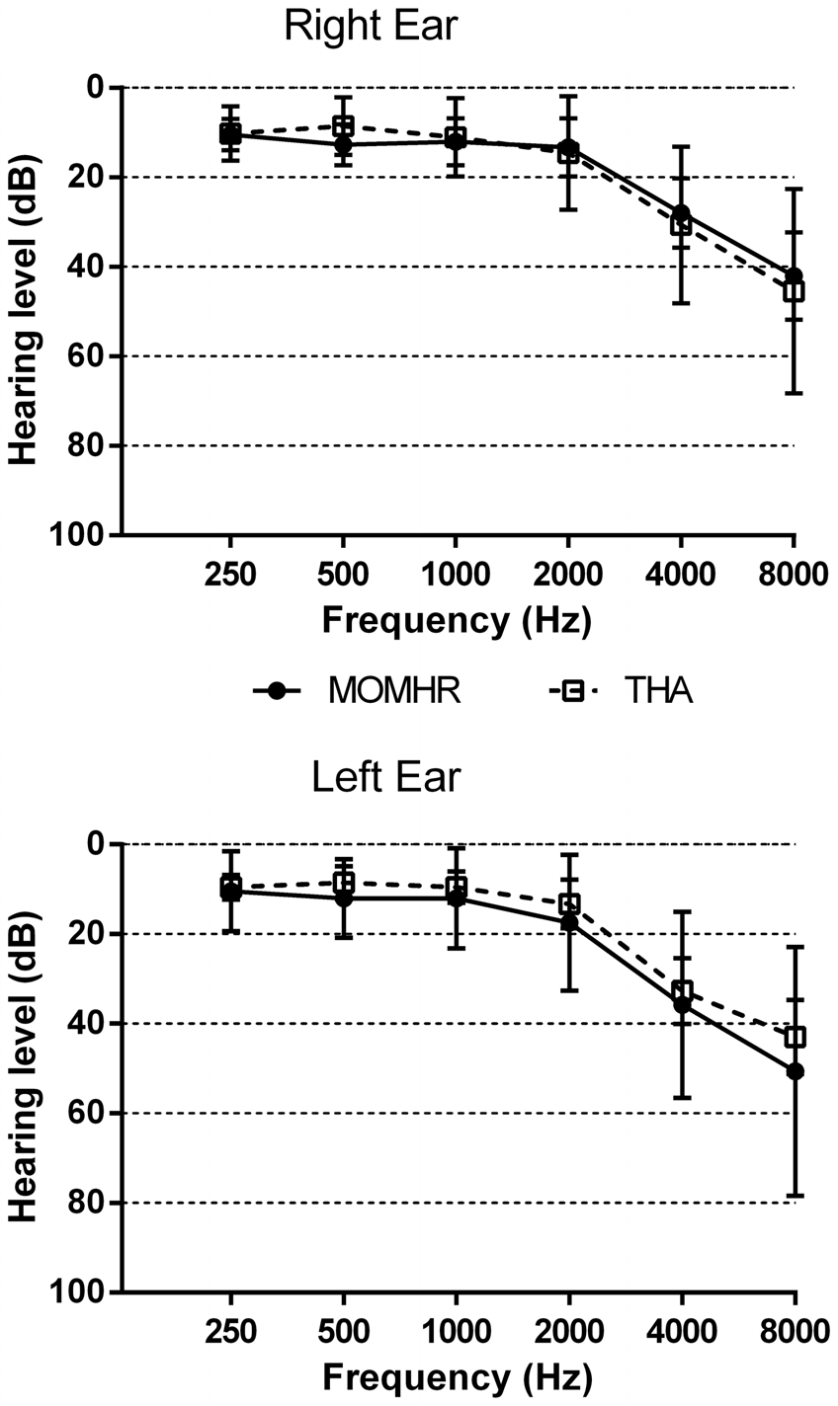
Pure Tone Audiogram hearing thresholds in MoMHR versus THA patients for (top panel) left and (bottom panel) right ears. Data are mean (95% confidence interval) hearing level threshold (dB). Analyses are MoMHR versus THA groups by Mann-Whitney test at each frequency, P>0.05 all comparisons.

The PTA findings were similar between groups (Figure 1, P>0.05 for all comparisons). The median half peak levels for speech discrimination in the subjects best ear were 19 dB (interquartile range 13 to 24) in the MoMHR subjects versus 17 dB (14 to 20) in the THA subjects (P = 0.56). The median half peak levels for speech discrimination expressed as the average of both ears were 21 dB (17 to 26) in the MoMHR subjects versus 20 dB (16 to 24) in the THA subjects (P = 1.00).

#### Objective audiometric tests

**Figure 2:**
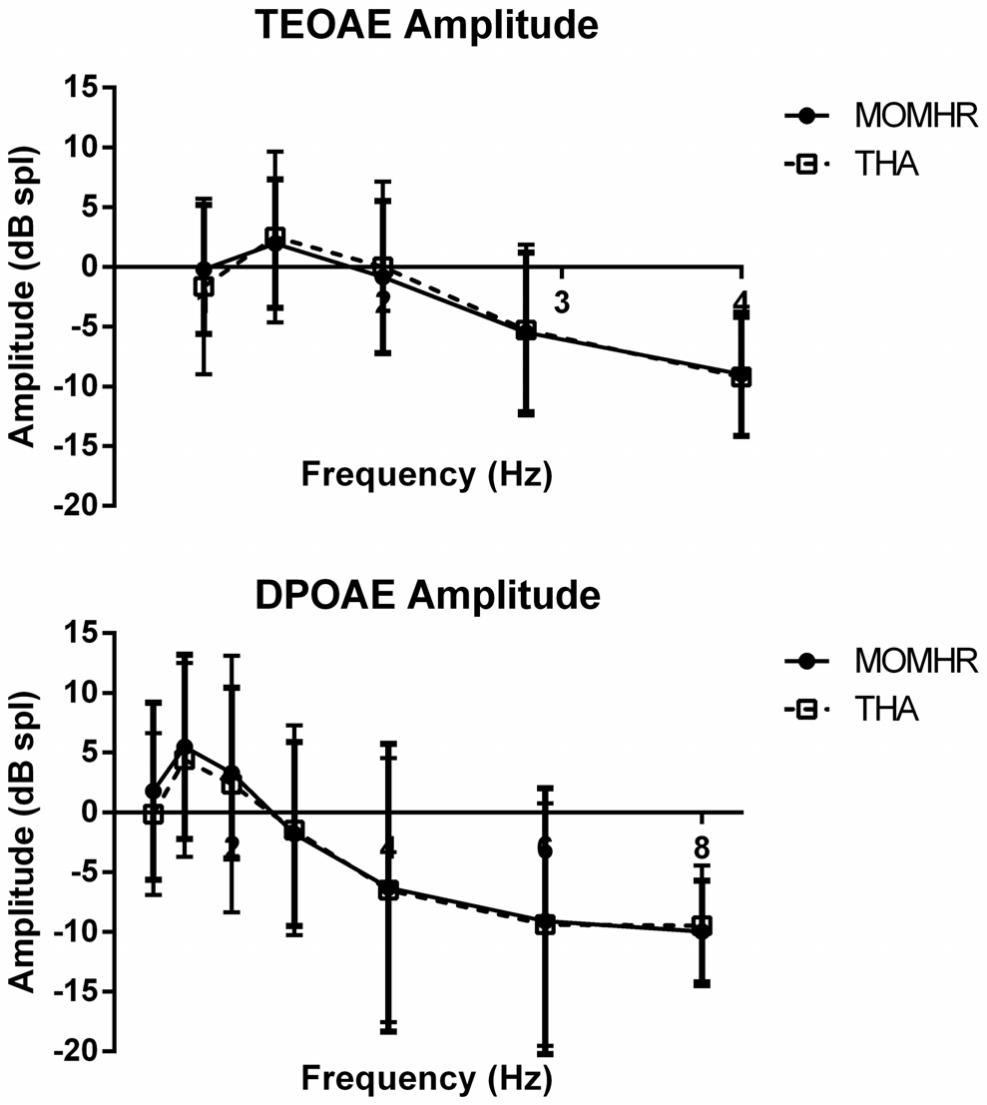
Transient Evoked Otoacoustic Emissions (TEOAE, top panel), Distortion Product Otoacoustic Emissions (DPOAE, bottom panel). Graphs show mean and standard deviation of the amplitude recorded for each of the frequencies tested. Analysis Students T-test, P>0.05 for all.

The amplitude and signal-to-noise ratio of the otoacoustic emissions (TEOAE and DPOAE) for all the frequencies tested were similar between groups (Figure 2, P>0.05 all comparisons). When considering an emission to be present when the signal-to-noise-ratio is greater than 6 dB and the signal greater than −5 dB, the percentage of subjects gaining a positive test result were also similar. At higher frequencies fewer emissions were recorded for both groups. Auditory brainstem responses, expressed as latency time of wave V and the inter-aural latency differences were similar between the two groups (Table 3, P>0.05 for both comparisons). No difference was observed between the ACR parameters measured. The latency times of N1 and P2 peaks as well as the N1-P2 latency and amplitude were all similar (Table 3, P>0.05 for all comparisons).

**Table 3:** Auditory responses.

Variable	MOMHR (n = 24)	THA (n = 24)
ABR Wave V latency (L-R) (ms)	0 (−0.06 to 0.12)	0.08 (−0.05 to 0.17)
ABR Wave V latency Mean (ms)	5.77 (5.62 to 5.93)	5.97 (5.74 to 6.09)
CERA N1 Latency (ms)	106.0±6.0	106.4±5.4
CERA P2 Latency (ms)	189.5±21.6	184.4±20.1
CERA N1-P2 latency (ms)	83.5±19.0	78.0±19.3
CERA N1-P2 amplitude (µV)	11.6±3.2	10.2±3.3

Values are mean ± standard deviation or median (interquartile range). Analyses are MoMHR versus THA by students t-test or Mann Whitney test; P>0.05, all comparisons. ABR = Auditory Brainstem Response, CERA = Cortical Evoked Response Audiometry.

### Visual outcomes

#### Clinical visual evaluation

Unilateral primary ocular disease was identified in 8 participants. In the MOMHR group there were 2 subjects with amblyopia and 1 subject with a previous penetrating eye injury. In the THA group there were two 2 subjects with previous eye trauma, 1 subject with a previous retinal detachment, 1 subject with amblyopia and 1 subject with severe macular degeneration. These participants were excluded from the binocular tests of visual and contrast acuity, Farnsworth-Munsell 100-Hue Test and the NEI-VFQ. For the OCT, which was performed on individual eyes, only the affected eye was excluded.

#### Visual symptoms

Patients in both groups had similar perceived visual functioning quality assessed by the NEI-VFQ and no evidence of neuro-ophthalmologic dysfunction using the neuro-ophthalmic supplement (Table 4, P>0.05 for all comparisons).

**Table 4:** Visual function.

Test	MOMHR (n = 21)	THA (n = 19)
VFQ: 25 standard questions	98.2 (95.8 to 98.2)	97.4 (94.4 to 98.2)
VFQ: Neuro-ophthalmic supplement	97.5 (87.5 to 100)	97.5 (87.5 to 100)
Binocular visual acuity (LogMar)	−0.06 (−0.17 to −0.05)	−0.08 (−0.14 to 0.00)
Contrast visual acuity (LogMar): 2.5%	0.22 (0.20 to 0.33)	0.26 (0.22 to 0.42)
Contrast Visual Acuity (LogMar): 1.25%	0.44 (0.40 to 0.60)	0.42 (0.38 to 0.52)
Color discrimination: Total error score (TES)	52 (40 to 82)	80 (52 to 108)
Color discrimination: 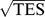	7.58±2.29	9.10±2.50
Color discrimination: Blue-yellow error score	32 (19 to 56)	39 (27 to 58)
Color discrimination: Red-green error score	27 (19 to 38)	30 (13 to 55)

Values are median (interquartile range). Analysis is MoMHR versus THA by Mann Whitney test; P>0.05, all comparisons. VFQ = Visual Functioning Questionnaire.

#### Subjective visual assessments

Both groups had similar logMAR binocular visual acuity and similar low contrast visual acuity (at 2.5% and 1.25%) (Table 4, P>0.05 all comparisons). Colour discrimination using the Farnsworth-Munsell 100-Hue test was similar between the two groups (Table 4, P>0.05). Both groups also showed poorer blue-yellow colour discrimination ability compared with red-green, consistent with the age-related decline in colour discrimination (Table 4, P>0.05).

#### Objective visual assessments

**Figure 3:**
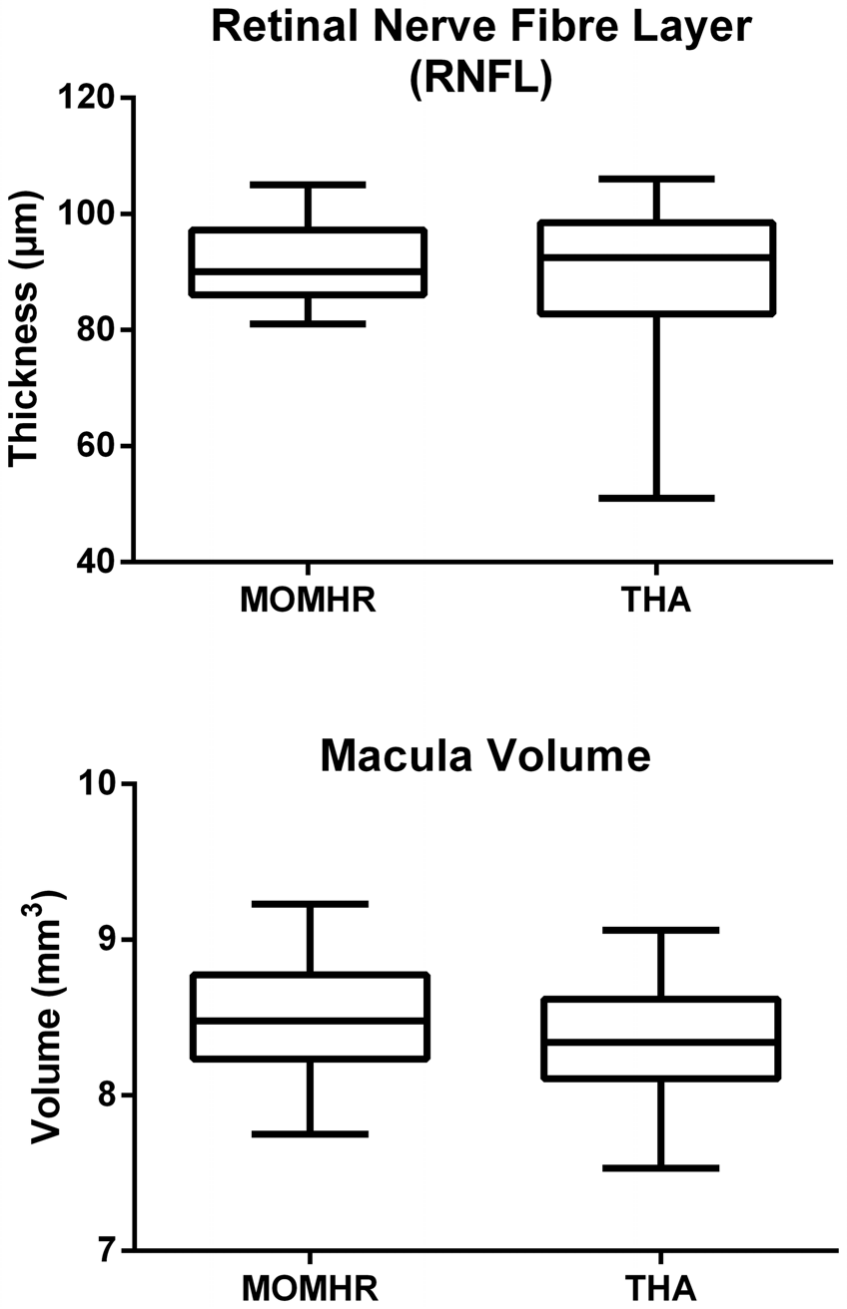
Box and whisker plots showing (top panel) difference in retinal nerve fibre layer thickness, and (bottom panel) differences in macular volume between the patient groups. All participants (n = 24) included as either non-pathological eye only or mean of right and left. All analyses are MOMHR versus THA by independent t-test (P>0.05 for both comparisons).

Both RNFL thickness and macular volume measured by OCT was similar between groups (Figure 3, P>0.05 for both comparisons).

## Discussion

We studied visual and auditory health in patients chronically exposed to low levels of metal following MoMHR compared with age matched patients with the same duration of exposure to a conventional THA. This investigation followed the identification of structural differences in the brain between these patient groups [13], and aimed to determine whether these structural differences associated with quantifiable functional differences between the groups. Following exclusion of individuals with primary ocular or aural pathology, patients in both groups had similar auditory and visual function, as assessed by a range of clinical, subjective, and objective standardised tests.

This study was conducted in a clinically healthy population of patients. The MoMHR group is representative of the general MoMHR population, in that they are typically younger patients and male, and the majority received the most frequently used prosthesis brand, the Birmingham Hip Resurfacing (BHR). The patients also had well-functioning prostheses, representative of the majority of patients after MoMHR, reflected by good Oxford hip score and metal levels below the MHRA 7 µg/L threshold.

Case report and occupational exposure evidence suggests that exposure to high systemic levels of cobalt and chromium may result in damage to both the visual and auditory systems [6], [7], [8], [10], [11], [20]. Two of the case reports describe high frequency hearing loss in association with patient reported hearing loss [10], [11]. High frequency hearing loss is a normal feature of ageing in males (presbycusis), and this characteristic was present to a similar extent in both our patient groups. The further investigation of hearing loss by Rizzetti et al showed loss of auditory brainstem responses bilaterally [8]. Here we performed a much larger scale study and more detailed assessment and found no evidence of an association of MoMHR exposure with altered brainstem responses at metal levels experienced by the majority of patients. The investigation of individual cases of visual loss with high systemic metal levels has suggested optic nerve atrophy on funduscopy and optic nerve and tract hyper-intensity on MRI [8], [10]. Using the principle that damage to the optic nerve and tracts should be reflected in degeneration of the retinal ganglion cells we investigated retinal structure using OCT but found no differences between the groups in our population with well-functioning prosthesis.

It is important to note the differences between our study population and those patients described in the case studies. In contrast to those patients described in the case studies who had failing prostheses with very high metal levels (up to several hundred µg/L), our study was designed specifically to determine whether prolonged exposure to low elevated metal levels is associated with subtle defects.

Hip resurfacing remains a treatment option in the young male patient with arthritis. The current FDA advice refers to visual and auditory problems associated with systemic metal levels and advises close monitoring of high risk patients (FDA, 2013). Our data suggest that patients with chronic exposure to lower concentrations of circulating metal typical of well-functioning prostheses does not associate with clinically detectable defects in visual or auditory function. However, it remains unclear whether such effects may become clinically present with longer-term exposure.
